# Supplementary material for: Living with “long COVID”: A systematic review and meta-synthesis of qualitative evidence
Source: PLoS One. 2023 Feb 16;18(2):e0281884. doi: 10.1371/journal.pone.0281884 (PMC9934341; doi:10.1371/journal.pone.0281884)
Supplement: S2 File — (DOCX) [file pone.0281884.s002.docx]

***Supporting Information file 2: Quality appraisal of the included studies***

| Last name of the first author et al. (publication year) | Is there congruity between the stated philosophical perspective and the research methodology? | Is there congruity between the research methodology and the research question or objectives? | Is there congruity between the research methodology and the methods used to collect data? | Is there congruity between the research methodology and the representation and analysis of data? | Is there congruity between the research methodology and the interpretation of results? | Is there a statement locating the researcher culturally or theoretically? | Is the influence of the researcher on the research, and vice-versa, addressed? | Are participants, and their voices, adequately represented? | Is the research ethical according to current criteria or, for recent studies, is there evidence of ethical approval by an appropriate body? | Do the conclusions drawn in the research report flow from the analysis, or interpretation, of the data? |
| --- | --- | --- | --- | --- | --- | --- | --- | --- | --- | --- |
| Ladds et al. (2020) (24) | Y | Y | Y | U | Y | Y | N | Y | Y | Y |
| Kingstone et al. (2020) (23) | Y | Y | Y | Y | Y | N | N | Y | Y | Y |
| Buttery et al. (2020) (25) | Y | Y | U | Y | Y | N | N | U | Y | Y |
| Høier et al. (2021) (37) | Y | Y | Y | Y | Y | N | N | Y | Y | Y |
| Humphreys et al. (2021) (26) | Y | Y | Y | Y | Y | N | N | Y | Y | Y |
| Ladds et al. (2021) (27) | Y | Y | Y | Y | U | N | N | Y | Y | Y |
| Razai et al. (2021) (28) | Y | Y | Y | Y | Y | N | N | Y | Y | Y |
| Shelley et al. (2021) (29) | Y | Y | Y | Y | Y | N | N | Y | Y | Y |
| Taylor et al (2021) (30) | Y | Y | Y | Y | Y | N | N | Y | Y | Y |
| Burton et al (2022) (31) | Y | Y | Y | Y | Y | N | N | U | Y | Y |
| Callan et al. (2022) (32) | Y | Y | Y | Y | Y | N | N | Y | Y | Y |
| Day (2022) (33) (33) | Y | Y | Y | Y | Y | N | N | Y | Y | Y |
| Ireson et al. (2022) (34) | Y | Y | Y | Y | Y | N | N | Y | Y | Y |
| Khatri (2022) (35) | Y | Y | Y | Y | Y | N | N | Y | Y | Y |
| Schiavi et al. (2022) (36) | Y | Y | Y | Y | Y | N | N | Y | Y | Y |

Abbreviations: Y: Yes, N: No, U: Uncertain
