# Supplementary material for: Living with “long COVID”: A systematic review and meta-synthesis of qualitative evidence
Source: PLoS One. 2023 Feb 16;18(2):e0281884. doi: 10.1371/journal.pone.0281884 (PMC9934341; doi:10.1371/journal.pone.0281884)
Supplement: Attachment — Submitted filename: PONE-D-22-26432_review 4_12_2022.docx [file pone.0281884.s003.docx]

| Line | Article sections | Reviwer notes |
| --- | --- | --- |
|  | Abstract | No observations |
| 82-85 | Background/Introduction | In my opinion, a more accurate and detailed definition of what is meant by long covid is needed.  The following study should be considered for this purpose:   - Soriano, J. B., Murthy, S., Marshall, J. C., Relan, P., Diaz, J. V., & WHO Clinical Case Definition Working Group on Post-COVID-19 Condition (2022). A clinical case definition of post-COVID-19 condition by a Delphi consensus. *The Lancet. Infectious diseases*, *22*(4), e102–e107. https://doi.org/10.1016/S1473-3099(21)00703-9   Add a more detailed description of the health problems long covid patients had, specifying and listing what the organs/apparatus most affected are, and what the consequences are.  The following study should be considered for this purpose:   - Crook, H., Raza, S., Nowell, J., Young, M., & Edison, P. (2021). Long covid-mechanisms, risk factors, and management. BMJ (Clinical research ed.), 374, n1648. <https://doi.org/10.1136/bmj.n1648> - Michelen, M., Manoharan, L., Elkheir, N., Cheng, V., Dagens, A., Hastie, C., O'Hara, M., Suett, J., Dahmash, D., Bugaeva, P., Rigby, I., Munblit, D., Harriss, E., Burls, A., Foote, C., Scott, J., Carson, G., Olliaro, P., Sigfrid, L., & Stavropoulou, C. (2021). Characterising long COVID: a living systematic review. BMJ global health, 6(9), e005427. <https://doi.org/10.1136/bmjgh-2021-005427> |
|  | Material and methods | No observations |
| 177 -Table 2  189  212  372  445 | Results | Pag. 11 typing mistake: “Covid 19” / “~~a~~ fearful”  About the sample characteristics few details are given.  What was the average age? Were health workers included? How many patients have experienced a severe form and have been hospitalized?  In Table 3, the order of presentation of findings can be improved as shown in the attached pdf file, following an order of importance of physical problems. A different order of presentation of the categories that emerged is proposed.  “In several studies, many participants expressed concern regarding the availability and accessibility of personalized digital health support”.  Is the prevalent findings reference to the GP? if so, it would be helpful to clarify this further |
| 573 – table 9 | Discussion | Pag. 38 typing mistake: “Ensuring” |
