# Supplementary material for: Living with “long COVID”: A systematic review and meta-synthesis of qualitative evidence
Source: PLoS One. 2023 Feb 16;18(2):e0281884. doi: 10.1371/journal.pone.0281884 (PMC9934341; doi:10.1371/journal.pone.0281884)
Supplement: Attachment — Submitted filename: Response to Reviewers.docx [file pone.0281884.s005.docx]

1.23.2023

Dear Academic Editor and Reviewers,

Thank you for your valuable comments and feedback. These helped us to revisit our work and improve it substantially. We appreciate your responses and would like to thank you for giving us the opportunity to revise our work.

Please find the following responses and descriptions of changes made following your feedback.

***Responses to the reviewers***

| Comments of the reviewers | Our responses | Changes made to the manuscript |
| --- | --- | --- |
| It may be useful to define which apparatuses are most involved. (page 3) | Thank you very much for this comment. We have elaborated on this topic in the revised manuscript and added new information alongside appropriate citations. | Please see highlighted lines 72-76 in page 3 of the revised manuscript. |
| In my opinion, a more accurate definition of what is meant by long covid is needed. (page 3) | We appreciate this comment. In this revised submission, we have reflected more on the characteristics of long COVID. Also, we have cited all recommended citations and one additional review that describes the evolving evidence on long COVID. These new insights and added references will help the readers to follow recent scientific updates on the pathophysiology of long COVID. | Please see lines 74-81 on pages 3-4 of the revised manuscript. |
| Typing error- COVID-19 (page 11) | Thank you for bringing this to our notice. We have corrected this typing error. | Please see page 12 of the revised manuscript. |
| without "a" (page 12) | Thanks for mentioning this error- we have corrected it in the submitted version. | Please see page 13 of the revised manuscript. |
| Were health workers included? what was the average age? (page 13) | Thank you for your comment. The average age of the participants was not reported in all studies- most of them reported the age range. We mentioned that range in the revised version ensuring appropriate reporting. | Please see lines 198-199 on page 14 of the revised manuscript. |
| a different order of presentation of the categories that emerged is proposed (page 14) | Thank you very much for proposing this updated ranking. We agree that the updated ranking may help the readers to understand the severity of the symptoms. We have edited the table and arranged them as suggested by the reviewer. | Please see the updated Table 3 on pages 15-16 of the revised manuscript. |
| in several studies, many participants expressed concern regarding the availability and accessibility of personalized digital health support (page 25) | Thank you for suggesting this change- we have updated the text accordingly. | Please see lines 383-384 on page 28 of this revised submission. |
| given the model, is the prevalent reference to the GP? if so, it would be helpful to clarify this further (page 30) | Thanks for mentioning this issue. Most studies did not report GP-specific findings. Interestingly, studies from the EU countries specified the role of GPs alongside other HCPs. For this evidence synthesis, we included all of them and mentioned them more specifically to clarify this further. Thank you again for this suggestion. | Please see lines 457-458 on page 32 of the revised manuscript. |
| Is the relationship with healthcare professionals perceived equally by inpatients and outpatients? (page 30) | We appreciate this comment. As we mentioned in this review, most studies recruited participants from online support forums and diverse settings, making it difficult to understand the specific settings of care such as inpatients and outpatients. Very few participants reflected on their inpatient experiences, whereas most patients described their journey through healthcare without specifying specific points of care. For this reason, we mentioned all possible care facilities that may reflect the inclusiveness of opinions from study participants across settings. | Please see lines 462-463 on page 32 of the revised submission. |
| Is it known whether this phenomenon is more pronounced in transitions of care and hospitalized patients? (page 32) | Thank you for your comment. The study participants across reviews reported this phenomenon in the context of their experiences with long COVID, rather than mentioning specific phases of care, as we discussed above. We noticed that some quotes mentioned what happened within clinical settings, whereas many patients discussed their experiences during and after care transitions. We mentioned this specifically in the revised submission for better clarity. Thank you for this suggestion. | Please see line 489 in this revised manuscript. |
| “Ensuring” (page 38) | Thank you for bringing this error to our notice. We have edited and corrected this typo. Thank you again. | Please see page 40 of this revised submission. |
